# Author Correction: *h*-Analysis and data-parallel physics-informed neural networks

**DOI:** 10.1038/s41598-024-62284-9

**Published:** 2024-05-27

**Authors:** Paul Escapil‑Inchauspé, Gonzalo A. Ruz

**Affiliations:** 1 Facultad de Ingeniería y Ciencias, Universidad Adolfo Ibáñez, Santiago, Chile; 2 Data Observatory Foundation, Santiago, Chile; 3 Center of Applied Ecology and Sustainability (CAPES), Santiago, Chile

Correction to: *Scientific Reports* 10.1038/s41598-023-44541-5, published online 16 October 2023

The original version of this Article contained errors in section ‘General Notation’, Equation 3, Equation 13, Equation 14, Equation 21, Table 1, section ‘Proof Consider the setting of Theorem 1.’ and in section ‘Methodology’, where notations were incorrect.

In section ‘General Notation’,

$$T \vee : = card\left( T \right)$$

now reads:

$$T \vee : = \text{card}\left( T \right)$$

In Equation 3, 14, ‘Theorem 1’, ‘Proof of Theorem 1’:

$$u_{o} bs$$

now reads:

$$u_{obs}$$

In Equation 13, ‘Proof of Theorem 1’ and Equation 21:

$$C_{p} de$$

now reads:

$$C_{pde}$$

In section ‘Methodology’,

$$T^{t} est$$

now reads:

$$\mathcal{T}^\text{test}$$

In Table 1, in the heading of column 7:

$$N^{t} est$$

now reads:

$$N^{test}$$

The original Article has been corrected.

